# Potential Benefits of Limited Clinical and Radiographic Follow-up After Surgical Treatment of Ankle Fractures

**DOI:** 10.5435/JAAOSGlobal-D-21-00074

**Published:** 2021-05-11

**Authors:** Lisa G. M. Friedman, Daniela Sanchez, Terri A. Zachos, Andrew Marcantonio, Megan Audet, Heather Vallier, Brian Mullis, Adam Myers-White, Laurence Kempton, Jeffrey Watts, Daniel S. Horwitz

**Affiliations:** From the Geisinger Medical Center, Department of Orthopaedics, MSK Institute, Danville, PA (Dr. Friedman and Dr. Horwitz); the Universidad del Rosario, School of Medicine and Health Sciences, Bogota, Colombia (Dr. Sanchez); the Department of Orthopaedic Surgery, University of California Davis Health System, Sacramento, CA (Dr. Zachos); Department of Orthopedic Surgery, Lahey Hospital and Medical Center, Burlington, MA (Dr. Marcantonio); Department of Orthopaedic Surgery, Beaumont Health, Royal Oak, MI (Dr. Audet); Department of Orthopaedic Surgery, MetroHealth Medical Center, Case Western Reserve University, Cleveland, OH (Dr. Vallier); Department of Orthopaedic Surgery, Indiana University School of Medicine, Indianapolis, IN (Dr. Mullis); University of Miami, Department of Anesthesiology, Jackson Memorial Hospital, Miami, FL (Dr. Myers-White); Department of Orthopaedic Surgery, Carolinas Medical Center, Atrium Health Musculoskeletal Institute, Charlotte, NC (Dr. Kempton); and Department of Orthopedic Surgery, Cleveland Clinic Akron General Hospital, Akron, OH (Dr. Watts).

## Abstract

Ankle fractures are one of the most prevalent musculoskeletal injuries, with a significant number requiring surgical treatment. Postoperative complications requiring additional interventions frequently occur during the early postoperative period. We hypothesize that there is a limited need for routine clinical and radiographic follow-up once the fracture is deemed healed.

**Methods:**

IRB approval was obtained at four academic trauma centers. A retrospective chart review was done to identify adults with healed unimalleolar and bimalleolar ankle fractures treated surgically with at least 12 months of follow-up. Based on postoperative radiographs, changes in fracture alignment and implant position from radiographic union to final follow-up were documented. The average reimbursement for a final follow-up clinic visit and a set of ankle radiographs were estimated.

**Results:**

A total of 140 patients met inclusion criteria. The mean age at injury was 49.5 years, and 67.9% of patients were female. The mean time to healing was 82.2 days (±33.5 days). After radiographic healing, one patient had radiographic changes but was asymptomatic and full weight bearing at their final follow-up. On average, our institution was reimbursed $46 to $49 for a follow-up clinic visit and $364 to $497 for a set of ankle radiographs.

**Conclusion:**

Given the average time to healing, there is limited utility in routine radiographic and clinical follow-up beyond 16 weeks in asymptomatic patients. In our series, this would result in a savings of $950 to $1,200 per patient. However, after ankle fractures were deemed healed, 0.7% patients had radiographic evidence of a change in implant position. Documenting this change did not modify the immediate course of fracture treatment. Surgeons will need to balance the need for routine follow-up with the potential economic benefits in reducing costs to the healthcare system.

Ankle fractures are common injuries, occurring in 71 to 187 per 10,000 person years.^1,2,3,4,5,6^ Approximately 60% to 70% of ankle fractures are isolated unimalleolar fractures, whereas 10% to 20% of fractures are bimalleolar.^3,4^ The rate of ankle fractures has been increasing since the 1990s, with increased athletic participation and aging populations being contributing factors.^4,6^

Follow-up is an important component of postsurgical care. Radiographic and clinical follow-up can allow the treating surgeon to follow healing and clinical progress and monitor for complications, such as infection, nonunion, and loss of reduction. There is no consensus among orthopaedic surgeons as to how long diaphyseal, extra-articular metaphyseal, and intra-articular fractures should be followed clinically and radiographically.^7^ With the orthopaedic surgery community becoming more cost-conscious, the role and utility of routine postoperative imaging for a variety of fractures is the subject of ongoing research.^7,8,9,10,11^

Given the frequency of ankle fractures and operative fixation, minimizing radiographic and clinical follow-up that has no impact on patient management is a prime opportunity to reduce cost and improve access to care by freeing up surgeons to care for other patients. The purpose of this study was to determine the time to radiographic healing and follow changes in fracture alignment and implant position after healing has been achieved and estimate the economic costs associated with the clinical and radiographic follow-up.

## Methods

An institutional review board approval was obtained to conduct a retrospective study of patients surgically treated for ankle fractures at four level-I trauma centers between January 1, 2004, and December 31, 2015. Skeletally mature patients with internally fixed unimalleolar and bimalleolar ankle fractures with at least 12 months of follow-up were identified, and those patients in whom fracture union was documented were included in this study. Patients with posterior malleolar fractures, those undergoing syndesmotic fixation, and those who were neuropathic were excluded. In addition, patients who experienced nonroutine healing as defined by taking longer than 200 days to heal were excluded because the central study question focused on how long one needs to follow patients who exhibit clinical and radiographic healing, and it is already known that those who do not have healed fractures require prolonged follow-up.

Indications for surgical treatment included talar displacement, isolated malleolar displacement, or bimalleolar or bimalleolar equivalent fractures, open fractures, and fracture dislocations. A wide variety of surgical techniques were used to approach the fractures. The medial malleolus, when fixed, was done so using a lag screw, buttress plate, tension band, or hook plate. When fixed, the lateral malleolus was approached with a neutralization plate, bridge plate, buttress plate, or compression plate. All patients were immobilized after surgery, with the majority being placed in a splint. By week 2, most patients were transitioned into a cast or a boot. Patients were made partial weight-bearing on mean day 48.7 (SD 24.3) postoperatively and were made full weight-bearing on mean day 70.1 (SD 34.8) postoperatively.

No consensus exists among orthopaedic surgeons on how to assess fracture consolidation, and no radiographic scores have been validated for evaluating healing in ankle fractures. In this study, the definition of fracture healing was based on commonly used clinical and radiographic criteria, as described by Dijkman et al.^10^ A fracture was considered healed if a physician's note in the medical record stated the fracture was healed, or if there was radiographic evidence of callus formation, cortical continuity, loss of fracture line, and no tenderness to palpation over the fracture site.

We recorded patients' demographic information, fracture characteristics, time to healing, medical comorbidities, fixation method, weight-bearing status, secondary procedures, and number of follow-up visits and radiographs. Follow-up AP, lateral, and mortise ankle radiographs were reviewed, and changes in fracture alignment and implant position between radiographic healing and final follow-up were recorded. Fully trained orthopaedic research fellows measured and remeasured these radiographic changes at each of the sites. For patients with radiographic changes after radiographic healing was documented, the determination of symptoms was based on a review of office notes and the visual analog scale pain scores from clinic visits.

The amounts reimbursed to our institutions for a final clinic follow-up visit and a set of ankle radiographs were estimated using the average reimbursement from Medicare/Medicaid and randomly selected private insurers.

## Results

One hundred seventy-three surgically treated unimalleolar and bimalleolar ankle fractures with a minimum of 12 month follow-up were identified. Demographic and surgical data are presented in Table 1.

**Table 1:** Demographics, Fracture Characteristics, and Treatment

Demographics	
Age, mean	49.5 ± 16
Sex, N (%)	
Male	45 (32.1)
Female	95 (67.9)
Smoking, N (%)	37 (26.4)
Diabetes, N (%)	21 (15)
Fracture characteristics, N (%)	
Right	79 (56.4)
Left	61 (43.6)
Mechanism of injury, N (%)	
Fall	71 (50.7)
Twist	35 (25)
MVC	19 (13.6)
MCC	4 (2.9)
Sports	2 (1.4)
Other	7 (5.0)
Treatment	
Medial malleolus, N (%)	
Not fractured	32 (22.8)
Not fixed	21 (15)
Lag screw	60 (42.9)
Buttress plate	5 (3.6)
Tension band	2 (1.4)
Other fixation	5 (3.6)
Lateral malleolus	
Not fractured, N (%)	14 (10)
Lateral neutralization plate, N (%)	40 (28.6)
Lateral compression plate, N (%)	31 (22.1)
Lateral bridging plate, N (%)	12 (8.6)
Lateral buttress plate, N (%)	18 (12.9)
Posterolateral buttress plate, N (%)	19 (13.6)
Other fixation, N (%)	3 (2.1)
Time to surgery (d), mean	5.6 ± 5.4
Time to healing (d), mean	82.2 ± 33.5
Number healed by 6 wk, N (%)	8 (5.7)
Number healed by 9 wk, N (%)	38 (27.1)
Number healed by 12 wk, N (%)	80 (57.1)
Number healed by 16 wk, N (%)	121 (86.4)
Number healed by 20 wk, N (%)	129 (92.1)
Number healed by 25 wk, N (%)	137 (97.9)
Time to last follow-up (d), mean	991.6 ± 784.3

**Figure 1:**
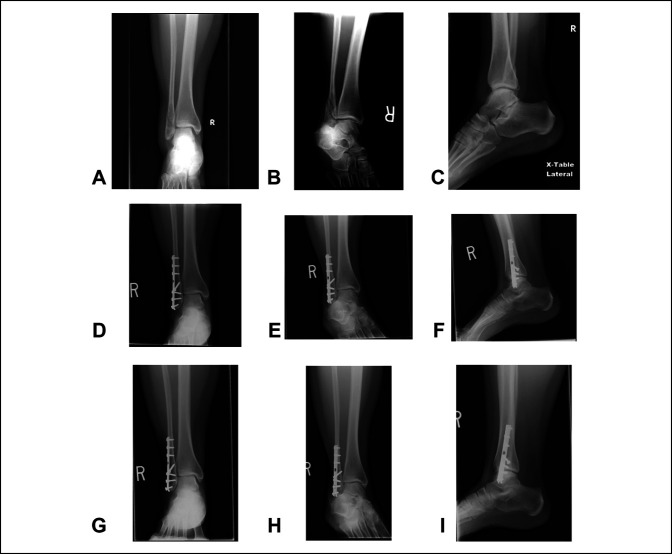
**A–C**, AP, mortise, and lateral preoperative radiographs demonstrating a distal fibula fracture. **D**–**F**, AP, mortise, and lateral radiographs approximately 7 months postoperatively demonstrating radiographic healing. **H**–**J**, AP, mortise, and lateral radiographs approximately 3.5 years postoperatively demonstrating backing out of the distal-most screw.

Ankle radiographs after the fracture was considered healed were reviewed. Twenty-eight (20%) had arthritic changes at the final follow-up. Twenty-four patients (17.1%) had symptomatic implants that were removed after fracture healing. The mean number of radiographs from healing to final follow-up was 2.3, and the mean number of clinic visits from healing to final follow-up was 2.9. After radiographic healing, in one patient (0.7%), backing up of the distal screw was documented 2 years after surgery (Figure 1). This patient was asymptomatic and was full weight-bearing at the final follow-up. No other patients experienced radiographic changes related to implant position or fracture alignment.

On average, our institution was reimbursed $46 to $49 for a follow-up clinic visit and $364 to $497 for a set of ankle radiographs.

## Discussion

Ankle fractures are very common injuries, accounting for up to 56% of fractures to the foot and ankle presenting to major trauma centers in the United States.^12^ The incidence of ankle fractures has been reported to be as high as 187 per 100,000 person years with the rate of fractures increasing over the past 30 years.^1,2,3,4,5,6^

After surgery, most patients are immobilized for a period of time and maintain a period of weight-bearing restriction, although recent literature has examined the safety and clinical outcomes of immediate weight-bearing as tolerated. The method of immobilization and length of time of weight-bearing restriction varies widely and is based on factors such as patient comorbidities, compliance, bone quality, and confidence in the fracture fixation.^13,14,15,16^ Patients are often followed up at 2, 6, 12, 24, and 52 weeks, but no standardized guidelines indicate when patients should be followed up clinically or radiographically.

Our current study demonstrated that once radiographic healing had been obtained, only one patient (0.7%) demonstrated a change in implant position, which did not alter immediate fracture management, although eventually the screw was removed. Once fracture union has been documented, additional radiographs provide no more notable information that modifies treatment of the fracture. Because the mean time to healing was 82.2 days (±33.5 days), there is limited utility to obtaining radiographs or clinical follow-ups in asymptomatic patients beyond 16 weeks. The benefits to limiting follow-up are substantial, including less radiation to the patient, convenience for the patient, and increased access for other patients who need orthopaedic care. In addition, based on our estimation of costs for radiographs and clinical follow-up, there would be a savings of $950 to $1,200 per patient by restricting clinical and radiograph follow-up once the fracture is healed.

Notably, many patients were still in need of orthopaedic care relating to their ankle after their fractures had healed. Indeed, 28 (20%) patients had arthritic changes at the final follow-up. Twenty-four patients (17.1%) had symptomatic implants that were removed after fracture healing. Thus, although our study demonstrates limited utility in routine follow-up in asymptomatic patients once healing has been achieved, symptomatic patients may still require follow-up, particularly if an intervention is being entertained. In our practice, we have found that symptomatic patients will seek follow-up without scheduled appointments. Thus, although mandatory follow-up is unnecessary after radiographic healing, clear communication about the availability to follow up as needed is paramount. Future studies are needed to determine to what extent radiographic and clinical follow-ups are needed throughout the entirety of caring for ankle fractures—from before radiographic healing has been achieved to monitoring for post-traumatic sequelae.

Previous literature has drawn the need for routine radiographic and clinical follow-up in asymptomatic patients into question. Harish et al. retrospectively reviewed 30 patients treated operatively for malleolar fractures and found in 25 of these cases that the postoperative formal radiographs were identical to the intraoperative fluoroscopic images, and in all cases, the radiographs were within normal limits for all measurements, indicating a limited role for radiographic follow-up.^17^ Similarly, Miniaci-Coxhead et al^18^ compared formal postoperative imaging before discharge with intraoperative fluoroscopic imaging for operatively treated ankle fractures and found that no formal postoperative imaging changed the treatment plan and formal radiographs were of poorer quality because of splinting material and poor rotation. Similarly, McDonald et al. compared 889 patients who received early postoperative imaging (7 to 21 days) with 522 patients who received late postoperative imaging (22 to 120 days) for operatively treated ankle fractures. No difference was observed in the rate of complications between the two groups, suggesting, in the absence of clinical suspicion, limited role for prolonged radiographic follow-up.^19^ van Gerven et al^20^ reviewed 936 routine postoperative ankle radiographs and found that this imaging results in changing the treatment strategy only 1.2% of the time.

The strengths of our study are the large sample size, the multisite study design, the range of techniques for fracture fixation, and the 12 months of follow-up for all patients, allowing for a broad understanding of the natural history of surgically treated ankle fractures. This study also had limitations intrinsic to its nature as a retrospective study. The definition of fracture healing remains subjective and union was judged using nonvalidated criteria. In addition, the number of clinical visits and postoperative radiographs were not standardized, meaning we could only estimate the cost savings from limiting routine follow-up after fracture healing had been achieved. Surgical indications and surgical techniques were also not standardized. Although conceivably this may affect the rate of complications and outcomes, it allows us to draw a more robust conclusion about the necessity of routine follow-up after healing has been achieved, regardless of factors relating to the surgery.

Ankle fractures are common injuries with an incidence that is increasing. Limiting routine radiograph and clinical follow-up in asymptomatic patients once healing has been achieved is safe and effective. Following these patients on an as-needed basis confers considerable benefit, including decreased cost, radiation exposure, and increased convenience.
